# Assessing Improvement in Prescription Writing Quality in Adult Male and Female Wards of Psychiatry Department, Lahore, Pakistan: Three Cycles of Clinical Audit

**DOI:** 10.1192/bjo.2024.629

**Published:** 2024-08-01

**Authors:** Aazeen Khan, Umaira Arif, Aamir Shahzad, Sania Mumtaz Tahir, Roop Kiran Khan

**Affiliations:** King Edward Medical University, Lahore, Pakistan

## Abstract

**Aims:**

1. To measure the extent to which medication orders in inpatient prescription charts conform to the section in BNF (British National Formulary) on prescription writing.
2. To implement changes with the intention of improving prescriptions and administration records.

**Methods:**

Prescription charts of patients admitted in adult male and female psychiatry ward were analysed in three cycles, (1 September to 20 October 2022, then 1st December to 31st 2022 and then: 1st January 2023 to 28th February 2023) which added up to a total of 431, 170 and 490 prescriptions in respective cycles.

Each drug prescription was examined to see if it met the standards outlined in BNF.

Percentage of prescriptions meeting each standard was calculated in each cycle.

First Cycle was followed by presentation of BNF guidelines of prescription writing on 7th December 2022 and copies of those BNF guidelines were placed at both male and female nursing counters. After 1 month, a short re-audit was done to assess the improvement which was satisfactory but this audit's results were not presented. Lastly, after one year of presentation of BNF guidelines in the department, two months of prescription charts were re-audited in cycle 3.

**Results:**

- Cycle 1: Initial evaluation revealed significant discrepancies in prescription accuracy and adherence to administration protocols. Key areas for improvement were identified and discussed with the postgraduate residents.
- Cycle 2: Following the implementation of targeted interventions, a re-evaluation showed measurable improvements in prescription accuracy and compliance with administration protocols. However, areas for further improvement were still identified, particularly in the documentation of prescription changes.
- Cycle 3: The final cycle demonstrated further improvements in prescription practices, with a significant reduction in discrepancies and errors.
- Legibility remained high across all cycles, with a slight improvement in Cycle 3.
- The use of generic drug names saw a remarkable increase from 40.6% in Cycle 1 to 84.69% in Cycle 3, indicating a strong adherence to best practices.
- Block letters usage improved significantly from 17% in Cycle 1 to 71.42% in Cycle 3, enhancing the clarity of prescriptions.
- The practice of providing a start date saw near-perfect compliance by Cycle 3, increasing from 82.8% in Cycle 1 to 99.18%.

Other findings were similar as well.

**Conclusion:**

The audit successfully demonstrated the effectiveness of clinical audits in improving prescription quality in male and female adult wards. It highlighted the effectiveness of the interventions and the importance of continuous monitoring and feedback.

